# 43017 Learning about Adaptive Capacity and Preparedness of CTSA Hubs

**DOI:** 10.1017/cts.2021.583

**Published:** 2021-03-30

**Authors:** Boris Volkov, Bart Ragon, Jamie Doyle, Miriam A. Bredella, Sandra Burks, Gaurav Dave, Keith Herzog, Kristi Holmes, Veronica Hoyo, Joe Hunt, Cathleen Kane, Wayne T. McCormack, Tanha Patel, Chris Pulley, Anne Seymour, Raj C. Shah, Laura Viera, Anita Walden

**Affiliations:** 1 University of Minnesota Clinical and Translational Science Institute; 2 Integrated Translational Health Research Institute of Virginia; 3 National Center for Advancing Translational Sciences, National Institutes of Health; 4 Massachusetts General Hospital and Harvard Medical School; 5 University of Virginia, Integrated Translational Health Research Institute of Virginia; 6 North Carolina Translational & Clinical Sciences Institute; 7 Northwestern University Clinical and Translational Sciences Institute; 8 University of California, San Diego Clinical and Translational Research Institute; 9 Indiana University Clinical and Translational Sciences Institute; 10 NYU Langone’s Clinical and Translational Science Institute; 11 University of Florida Clinical and Translational Science Institute; 12 Johns Hopkins University ICTR; 13 Rush University, Institute for Translational Medicine; 14 University of North Carolina at Chapel Hill, Translational & Clinical Sciences Institute; 15 Oregon Clinical and Translational Research Institute

## Abstract

ABSTRACT IMPACT: This work will inform the ongoing development of adaptive capacity and preparedness of the CTSA Program and other clinical and translational research organizations in their quest of improving processes that drive outcomes and impacts, shaping effective programs and services, and strengthening their emergency readiness and sustainability. OBJECTIVES/GOALS: -Share the progress and preliminary findings of an ‘Adaptive Capacity and Preparedness of CTSA Hubs’ CTSA Working Group; -Improve our awareness and understanding of the efficient and effective changes helping CTSA hubs build robust capacity to address METHODS/STUDY POPULATION: A multi-case study including: - Triangulating multiple sources of information and mixed methods (survey/interviews of research administrators, researchers, evaluators, and other key stakeholders), literature review, document and M&E system information analysis, and expert review; - Describing CTSA hubs’ experiences as related to research implementation, translation, and support during the time of emergency; - Administering a comprehensive survey of the CTSAs addressing their challenges, lessons learned, and practices that work in various program components/areas. Data collection includes aggregate and cross-sectional data, with representation based on CTSA size, maturity, and population density. RESULTS/ANTICIPATED RESULTS: The described approach shows sound promise to investigate and share strategies and best practices for building adaptive capacity and preparedness of CTSAs -- across various scientific sectors, translational research spectrum, and the goals outlined by NCATS for the CTSA program. The anticipated results of this research will include the identified/shared innovative solutions and lessons learned for this rapidly emerging, high-priority clinical and translational science issue. ‘High-quality lessons learned’ are those that represent principles extrapolated from multiple sources and triangulated to increase transferability to new contexts and situations. DISCUSSION/SIGNIFICANCE OF FINDINGS: The project provides useful knowledge and tools to research organizations and stakeholders across multiple disciplines -- for mitigating the impact of the COVID-19 disaster via effective adjusting programs, practices, and processes, and building capacity for future successful, ‘emergency ready and responsive’ research and training.

